# Quantification of myocardial fibrosis by digital image analysis and interactive stereology

**DOI:** 10.1186/1746-1596-9-114

**Published:** 2014-06-09

**Authors:** Dainius Daunoravicius, Justinas Besusparis, Edvardas Zurauskas, Aida Laurinaviciene, Daiva Bironaite, Sabine Pankuweit, Benoit Plancoulaine, Paulette Herlin, Julius Bogomolovas, Virginija Grabauskiene, Arvydas Laurinavicius

**Affiliations:** 1 Vilnius University Medical faculty, Department of Pathology, Forensic Medicine and Pharmacology, M. K. Ciurlionio 21/27, Vilnius 03101, Lithuania; 2 National Center of Pathology, Affiliate of Vilnius University Hospital Santariskiu Klinikos, Vilnius, Lithuania; 3 Department of Stem Cell Biology, Center for Innovative Medicine, State Research Institute, Vilnius, Lithuania; 4 Department of Cardiology, University Hospital Giessen & Marburg, Marburg, Germany; 5 Path-Image/BioTiCla, University of Normandy, Unicaen, Caen, France; 6 Medical Faculty Mannheim, University of Heidelberg, Mannheim, Germany

**Keywords:** Cardiac, Fibrosis, Quantification, Digital, Stereology

## Abstract

**Background:**

Cardiac fibrosis disrupts the normal myocardial structure and has a direct impact on heart function and survival. Despite already available digital methods, the pathologist’s visual score is still widely considered as ground truth and used as a primary method in histomorphometric evaluations. The aim of this study was to compare the accuracy of digital image analysis tools and the pathologist’s visual scoring for evaluating fibrosis in human myocardial biopsies, based on reference data obtained by point counting performed on the same images.

**Methods:**

Endomyocardial biopsy material from 38 patients diagnosed with inflammatory dilated cardiomyopathy was used. The extent of total cardiac fibrosis was assessed by image analysis on Masson’s trichrome-stained tissue specimens using automated Colocalization and Genie software, by Stereology grid count and manually by Pathologist’s visual score.

**Results:**

A total of 116 slides were analyzed. The mean results obtained by the Colocalization software (13.72 ± 12.24%) were closest to the reference value of stereology (RVS), while the Genie software and Pathologist score gave a slight underestimation. RVS values correlated strongly with values obtained using the Colocalization and Genie (r > 0.9, p < 0.001) software as well as the pathologist visual score. Differences in fibrosis quantification by Colocalization and RVS were statistically insignificant. However, significant bias was found in the results obtained by using Genie versus RVS and pathologist score versus RVS with mean difference values of: -1.61% and 2.24%. Bland-Altman plots showed a bidirectional bias dependent on the magnitude of the measurement: Colocalization software overestimated the area fraction of fibrosis in the lower end, and underestimated in the higher end of the RVS values. Meanwhile, Genie software as well as the pathologist score showed more uniform results throughout the values, with a slight underestimation in the mid-range for both.

**Conclusion:**

Both applied digital image analysis methods revealed almost perfect correlation with the criterion standard obtained by stereology grid count and, in terms of accuracy, outperformed the pathologist’s visual score. Genie algorithm proved to be the method of choice with the only drawback of a slight underestimation bias, which is considered acceptable for both clinical and research evaluations.

**Virtual slides:**

The virtual slide(s) for this article can be found here: http://www.diagnosticpathology.diagnomx.eu/vs/9857909611227193

## Background

Cardiac fibrosis is associated with disruption of the normal myocardial structure by excessive deposition of extracellular matrix. The term fibrosis encompasses several processes including fibroblast proliferation, collagen synthesis and degradation, as well as conversion of fibroblasts into a contractile “myofibroblast” phenotype. Myocardial matrix remodeling and fibrosis appear to play a pivotal role in the development of ventricular dilatation and heart failure [1]. The net effect of cardiac fibrosis is exaggerated by the increased tissue stiffness, impaired contraction due to myocyte slippage (separation), disrupted electrotonic connectivity and tissue hypoxia [2]. For these reasons, it is particularly important to understand cardiac fibrosis as the mechanistic basis of cardiac remodeling. It is well known, that fibrosis and certain histological changes in the myocardium impact heart function and even survival [3,4].

Evaluation of the extent of fibrosis, including semi-automated and semi-quantitative methods has been introduced earlier, however, detailed literature on methodological and technical aspects of quantification of fibrosis is scarce [5]. Most previous studies explored liver and kidney fibrosis [6-10], but up to now only a few have attempted to automatically quantify cardiac fibrosis [11-17]. Another limitation of recent publications is that the evaluations of cardiac fibrosis mostly have been done on animal models (mice, rats, dogs, pigs) and only few studies are on human hearts [18,19]. Moreover, the majority of such studies lack data validation to an appropriate criterion standard and the reference values are obtained by semi-quantitative visual evaluations rather than by more direct quantitative estimates.

Significant drift towards automation and quantification in pathology has occurred during the last decade [20-22]. Digital imaging in pathology provides users with similar functionalities of a microscope, but with numerous additional benefits and consequently, replaces subjective visual evaluation by presumably more objective and reproducible digital analyses [23-26]. Several applications of image analysis have recently received clearance from US Food and Drug Administration, indicating that automated quantification may provide more reliable and reproducible results than visual evaluation [20,27]. Numerous recent studies show that advanced computer image analyses can be successfully introduced in clinical practice and research [28-30]. Meanwhile, the interpretation of histomorphometric parameters in clinical routine and research is still primarily based on human visual scoring, which is hugely subjective [25,26]. Many factors affect human vision including: contrast, borders and color – all these impacts may be easily illustrated using a number of optical illusions. Semi-quantitative scoring not only involves a substantial workload on a pathologist, but also has several limitations inherent to the traditional pathology, such as significant intra- and inter-observer variation along with low efficiency [31].

Segmentation of stained tissue images is a complex problem, because of a large variability of the tissue samples (shape, size, color and architecture) [32]. Growing numbers of virtual slides that must be processed, transmitted and analyzed create a clear need of additional image correction and standardization algorithms [33]. Automatic selection of slides, application of appropriate thresholds and also a reliable selection of the slide areas containing the most significant information (regions of interest (ROI)) to deriving the diagnosis is becoming of major importance in virtual pathology [34]. Only a complete set of these computerized algorithms can eventually replace the pathologist’s unique work [22,35].

The most common practice of implementing a new digital algorithm is to compare the results obtained with the pathologist’s visual evaluation, that is, to validate it against the best clinically accepted method. This perception, however, is no longer valid: why should one calibrate a potentially more accurate and precise tool against a variable and semi-quantitative evaluation method? To estimate the accuracy of a new method, a criterion standard has to be obtained from an independent source measured in the most possible objective way. In this regard, stereology grid count, rather than the pathologist’s visual impression should be used [36-38]. Therefore, we performed our study on evaluating the accuracy of digital image analysis tools and the pathologist’s visual scoring for the measurement of fibrosis extent (ie: area fraction) in human myocardial biopsies, based on reference data obtained by point counting performed on the same images.

## Methods

### Experimental model

**Figure 1:**
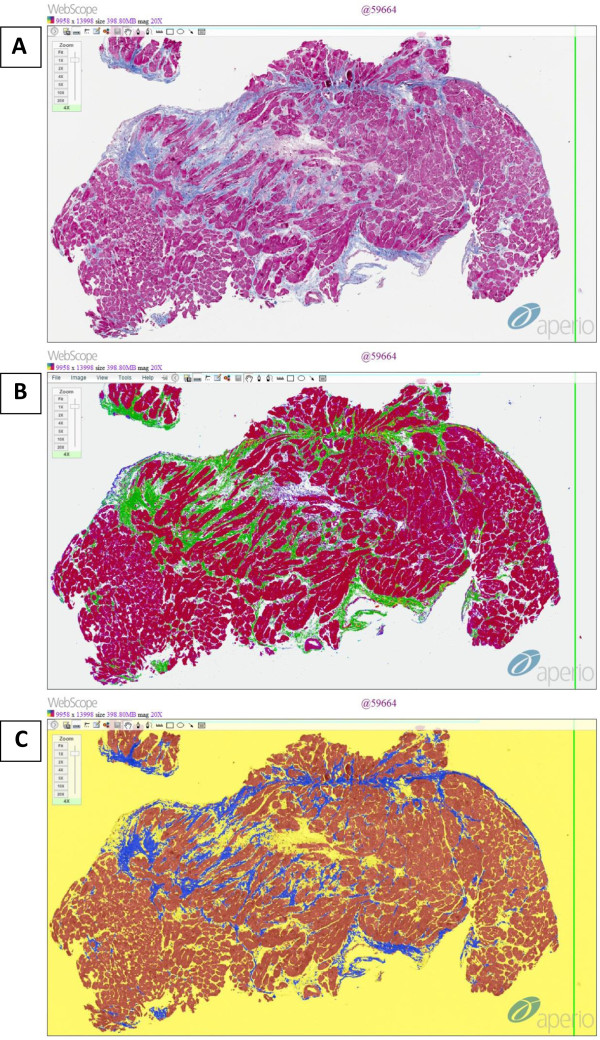
**Fibrosis mark-up on digitized slide: (A) Masson trichrome original staining, (B) Colocalization algorithm, (C) Genie algorithm.**

The study was conducted on endomyocardial biopsy (EMB) material from 38 patients (29 males, 9 females, mean age 42.3 ± 12.2 years) diagnosed with inflammatory dilated cardiomyopathy. All EMB specimens were collected between July, 2010 and February, 2013. Before EMB, each patient underwent coronary angiography to exclude coronary artery disease. Right ventricular EMB was obtained using a flexible bioptome via the right femoral vein [39]. At least 3 EMBs were subjected to histological evaluation. All specimens were included in the study to provide a full range of fibrosis.Tissue samples for histological analysis were fixed in 10% neutral buffered formalin with subsequent routine paraffin embedding. 3 μm-thick sections were used through the study. Sections were stained with Masson’s trichrome according to a standard protocol. Whole slide images (WSI) from the experimental glass slides were obtained at a resolution of 0.5 μm using a digital microscopic scanner (ScanScope® XT, Aperio Technologies, Vista, CA, USA) at a 20x objective magnification and stored in a tiled Tiff format on a devoted WSI server (Spectrum 11.1.0.751, Aperio) (Figure 1A). One section was later randomly chosen from the slide for all subsequent analyses. Aperio Colocalization and Genie algorithms were used for image analysis.

### Colocalization algorithm

Colocalization uses the color deconvolution [40] to separate the stains and classifies each pixel according to the number of stains present. For Colocalization, the threshold for each stain is specified for a required stain (e.g. Masson’s trichrome) and the algorithm reports the percentage of total tissue area for which each stain combination is detected: 1, 2, 3, 1 + 2, 1 + 3, 2 + 3, 1 + 2 + 3, or none (up to 3 stains are supported). The algorithm also provides an eight-color mark-up image for the visualization of the colocalized stains. The total percentage of cardiac fibrosis in biopsy samples was calculated according to the sums of the following stain combinations: 3, 2 + 3 and 1 + 3 (Figure 1B).

### Genie algorithm

**Figure 2:**
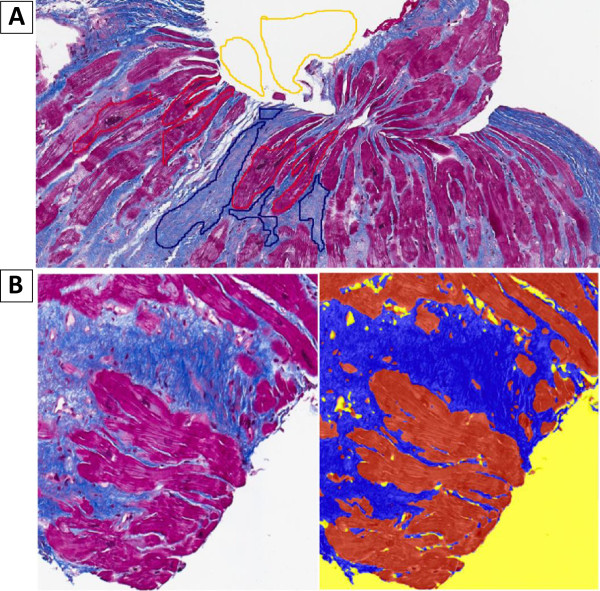
**Training and using of the genie: (A) Defining and marking the classes of interest,(B) Testing and using the new specific classifier.**

Genie (GENetic Imagery Exploration [41]) is a pattern recognition algorithm that distinguishes spatial and morphological features based on structures (classes) provided by the user. A specific Genie classifier was developed as follows: 1. New Genie project and training set created; 2. Digital slides added to a training set; 3. The classes of interest defined and marked in the digital slides in the training set (Figure 2A); 4. Training montage created by running Genie Training v1 algorithm (1000 training iterations set) on user-selected tissue sub-regions (the algorithm estimated the training accuracy at 99.4%); 5. Based on the training macro, Genie Classifier v1 algorithm was used to create the specific Classifier to be tested and used (Figure 2B). After testing the classifier the classes can then be selected for subsequent analysis using specific task algorithms. For better identification of cardiac fibrosis, we used only spatial recognition, disabling the detection of morphological features. For this study, the Genie system was trained to distinguish the myocardium, fibrous tissue (fibrosis) and glass (Figure 2B). Total cardiac fibrosis percentage was adjusted to a total tissue area in the image analyzed, ignoring the glass (Figure 1C).

### Stereology

**Figure 3:**
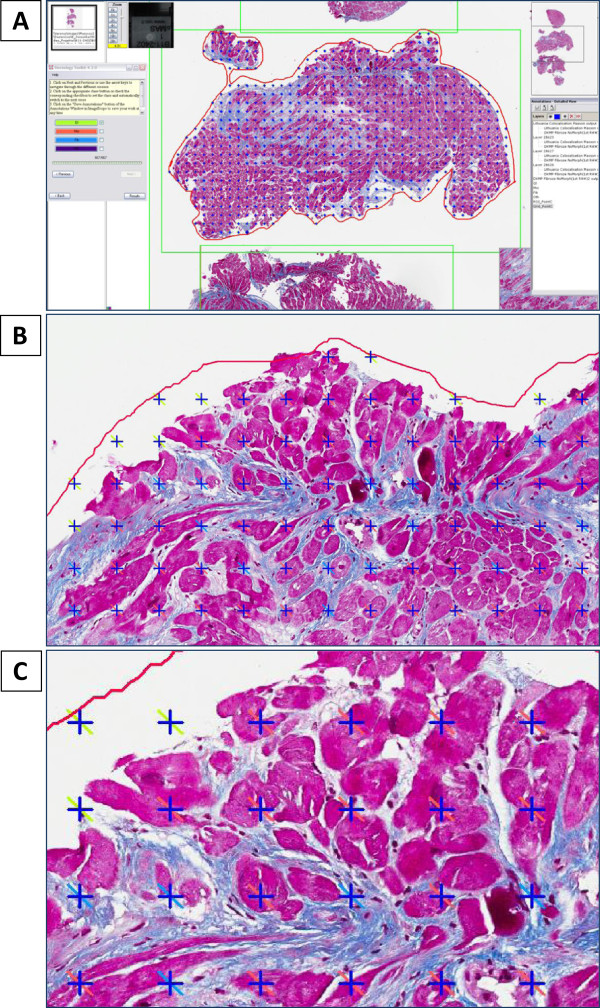
**Fibrosis mark-up on digitized slide using stereology protocol: (A) ImageScope V11 view incorporating grid (sampling step 200 pixels and size of the pattern 20 pixels), (B, C) structures of interest (glass, fibrosis, myocardium, other) manually highlighted by observer.**

Stereology is an interdisciplinary field for volume estimation of three-dimensional structures by their planar sections. We performed our study on tissue sections of 3 μm, thus the stereology was performed on a projection rather than on ideal 2D plane. A point counting grid was used to estimate the fraction area [42]. “Stereology toolkit 4.2.0” from ADCIS (Saint Contest, France) was used in this study. This stereology module allows defining a ROI and a grid that overlay an area of a virtual slide. Then the type, the spacing and the pattern size of the grid must be adjusted (Figure 3A). 150**–**200 test points are recommended for acceptable analysis precision [43,44]. The grid of point counting, with the sampling interval of 200 pixels and a pattern size of 20 pixels was chosen to evaluate the area fraction of myocardial tissue and cardiac fibrosis. These adjustments of the stereology grid ensured a minimum of 500 test points in the smallest myocardial biopsies and higher counting precision. The structures of interest: glass, fibrosis, myocardium, other (including inflammation, necrosis, glass areas inside the myocardium) were manually highlighted by the observer (Figure 3B, C). The total percentage of cardiac fibrosis was counted using the number of points ignoring the “glass” and “other” category. The area fraction, equivalent to the volume fraction of cardiac fibrosis was then estimated as the ratio between the number of test marked as fibrosis and the total number of test points included in the ROI, points ignoring the “glass” and “other” categories. The results were expressed as percentages together with the corresponding uncertainty computed according to Weibel [43].

### Pathologist’s visual scoring

The extent of total cardiac fibrosis in the samples was also evaluated as a percentage of the sample area by a highly-experienced pathologist using a light microscope. Two evaluations were performed with the time interval of two weeks.

### Statistical analysis

Data are presented as means and standard deviation (Mean ± std. deviation). For the statistical comparison of data, the Pearson’s correlation coefficient, Friedman’s test with post hoc (Wilcoxon signed-rank with a Bonferroni correction applied) and scatter-dot graphs (with r^2^, intercept and slope) were used. To enable a standard approach to the data, a natural logarithmic transformation of all measurements was performed before drawing scatted-dot plots. The agreement between fibrosis measurement methods was tested with Bland-Altman plots [45], using the stereology estimation as a reference method for the X axis [46]. All statistical analysis was performed using the SPSS package (version 19.0 for Windows; SPSS Inc., Chicago, IL, USA) at 5% significance level.

### Ethical approval

The study was approved by the Vilnius Regional Biomedical Research Ethics committee Nr.158200-09-382-103. All patients gave written informed consent to include their data in the study for each investigational procedure.

## Results

A total of 116 slides were analyzed digitally, by visual scoring and using stereology grids.

The mean result of fibrosis obtained by Colocalization software was 13.72 ± 12.24% being closest to the reference value of stereology (RVS: 13.21 ± 15.25%). The mean values obtained by the Genie software (11.60 ± 15.41%) and the pathologist’s score at week 0 (11.20 ± 15.53%) and week 2 (10.76 ± 17.37%) indicated a slight underestimation relative to RVS. However, the range of Colocalization software was 73.79% being the lowest of all tested methods with a difference of around 20%. The range of the Genie software was 88.22% and the pathologist’s score had the highest range of 100%. These results were comparable to the range of the RVS (96.50%), Table 1.

**Table 1:** Summary statistics for cardiac fibrosis (%) evaluation methods

	**Stereology**	**Colocalization**	**Genie**	**Pathologist week 0**	**Pathologist week 2**
Number of observations	116	116	116	116	116
Mean	13.21	13.72	11.60	11.20	10.76
Median	8.70	11.12	7.39	5.00	5.00
Std. deviation	15.25	12.24	15.41	15.53	17.37
Range	96.50	73.79	88.22	100.00	100.00
Minimum	0.00	1.57	0.05	0.00	0.00
Maximum	96.50	75.36	88.27	100.00	100.00

Both the Colocalization and Genie methods correlated very strongly with the RVS cardiac fibrosis estimates, yielding r = 0.928 and r = 0.946 (p < 0.001), respectively. Similarly, the pathologist’s visual score strongly correlated with RVS: r = 0.913 (p < 0.001) at week 0 and r = 0.929 (p < 0.001) at week 2 (Table 2).

**Table 2:** Pairwise correlations between stereology, digital algorithms and pathologist score (Pearson’s coefficients, p < 0.001, N = 116)

	**Stereology**	**Colocalization**	**Genie**	**Pathologist week 0**
Colocalization	0.928			
Genie	0.946	0.973		
Pathologist week 0	0.913	0.839	0.841	
Pathologist week 2	0.929	0.853	0.856	0.965

**Figure 4:**
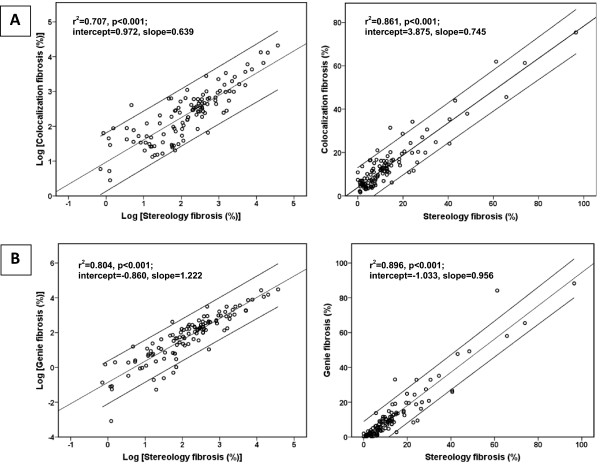
**Single linear regression models with reference values: (A) colocalization and stereology; (B) genie and stereology.** Linear regression line is presented within 95% confidence interval.

**Figure 5:**
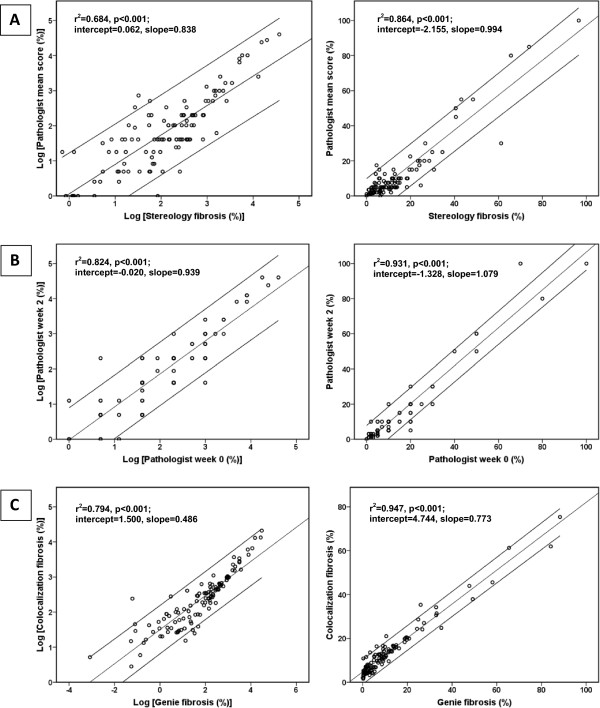
**Single linear regression models with reference values: (A) Pathologist mean score and stereology; (B) pathologist score at week 0 and week 2; (C) colocalization and genie.** Linear regression line is presented within 95% confidence interval.

**Figure 6:**
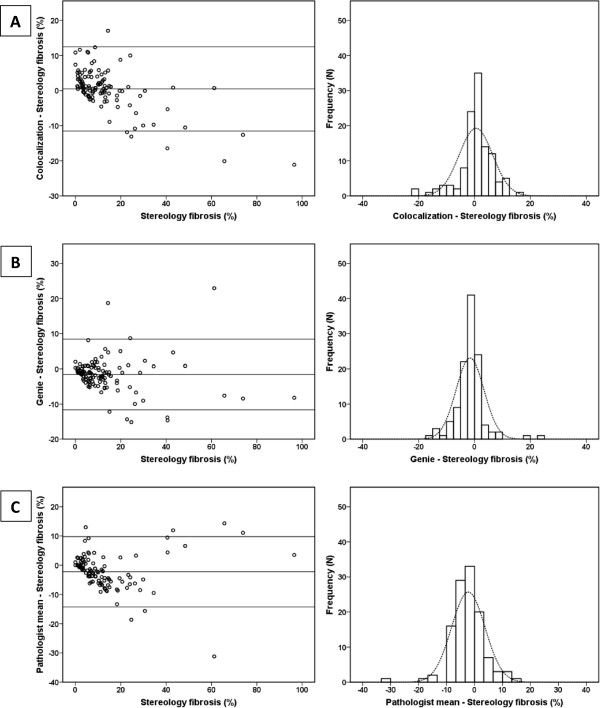
**Bland-Altman plots and histograms of the method score differences: (A) colocalization and stereology; (B) genie and stereology; (C) pathologist mean score and stereology.** Horizontal line represents mean difference within limits of agreement, which are defined as the mean difference ± 2 standard deviations.

Friedman’s test revealed statistically significant differences in the results of tested cardiac fibrosis evaluation methods *χ*^2^(3) = 62.405, p = 0.000. Post hoc analysis with Wilcoxon signed-rank tests with a Bonferroni correction (significance level set at p < 0.0125) was applied. The differences in the results of Colocalization versus RVS were statistically insignificant (Z = -2.259, p = 0.024) with a mean difference value of 0.50%. However, post hoc analysis showed significant differences between the results of Genie versus RVS (Z = -5.000, p = 0.000) and the pathologist’s mean score versus RVS (Z = -4.422, p = 0.000) with mean difference values of: -1.61% and 2.24%. Similarly significant difference of the results between both digital methods (Genie versus Colocalization) was noted: Z = -6.639, p = 0.000 with a variance bias of 2.11% (Table 3).Single linear regression model plots demonstrated some advantage of Genie software over the Colocalization software with noticeably better values in both original raw and log-transformed measurements for r-square 0.896 and 0.804 (log) versus 0.861 and 0.707 (log); slope 0.956 and 1.222 (log) versus 0.745 and 0.639 (log); intercept -1.033 and -0.860 (log) versus 3.875 and 0.972 (log) (Figure 4). The pathologist’s mean score correlation with RVS was similar: r-square 0.864 and 0.684 (log), slope 0.994 and 0.838 (log), intercept -2.155 and 0.062 (log); the inter-observer variation at week 0 and week 2 was negligible: r-square 0.931 and 0.824 (log), slope 1.079 and 0.939 (log), intercept -1.328 and -0.020 (log). Surprisingly, both digital methods did not correlate as well as expected with still acceptable r-square values (0.947 and 0.794 (log)), but high intercept (4.744 and 1.500 (log)) and slope far from ideal (0.773 and 0.486 (log)) (Figure 5).Bland-Altman plots showed a bidirectional bias dependent on the magnitude of the measurement: Colocalization software overestimated the area fraction of fibrosis in the lower end, and underestimated it in the higher end of the RVS scale (Figure 6A). Meanwhile, Genie software as well as the pathologist’s mean score showed more uniform results throughout the complete scale with a slight underestimation in the mid-range for both (Figure 6B, C). Presented histograms indicate a normal distribution of the differences for each plot (Figure 6).

**Table 3:** Paired comparison of cardiac fibrosis (%) evaluation methods

		**Paired differences**		Z*	p value*
		**Mean**	**Std. Deviation**		
Pair 1	Colocalization – Stereology	0.50	6.00	-2.259	0.024
Pair 2	Genie – Stereology	-1.61	5.02	-5.000	0.000
Pair 3	Pathologist mean – Stereology	2.24	6.01	-4.422	0.000
Pair 4	Colocalization – Genie	2.11	4.49	-6.639	0.000

* Based on post hoc analysis with Wilcoxon signed-rank tests (Bonferroni correction applied with significance level set at p < 0.0125).

## Discussion

To this day a pathologist’s visual score is widely accepted as ground truth and, despite already available digital methods, it is still used as a primary method for histomorphometric evaluations. Many attempts to incorporate digital methods into clinical practice face the same issue of proper validation – the digital analysis data are commonly compared to semi-quantitative visual evaluation, while most direct criterion standard yet requires time-consuming procedures.

The early study of Vasiljevic et al. [13] based on human endomyocardial biopsies compared results of semi-quantitative scoring, point-lesion counting (using a grid) to computer-assisted methods. This was the first study to demonstrate strong correlation of different cardiac fibrosis scoring methods, however, due to considerable input by the investigator in computer analysis it still can be considered as subjective to some degree. Particularly since a stereology test grid was not used for RVS. Hadi et al. [16] quantified cardiac fibrosis by automated analysis using ImageJ software and traditional polarization microscopy, with subsequent validation of the results, using stereology data as criterion standard. To our knowledge, it is the only study of cardiac fibrosis that applied stereology procedures to obtain RVS; however, the validation was performed on rat cardiac rather than human samples (the analysis was then tested on a post-mortem tissue samples from a 78 year old man).

In our study, we have tested several methods to evaluate the extent of human cardiac fibrosis, which can be readily implemented in clinical practice today. We used stereology as the most independent and objective RVS available and a modified Bland-Altman plot as the best statistical tool to measure agreement between the tested method and a RVS.

The initial data were somewhat in favor of the Colocalization software: it demonstrated the closest fibrosis mean value to a reference and resulting difference of 0.50% was statistically insignificant. However, the Colocalization software had a noticeably narrower variation, which was 20% behind the RVS and the pathologist’s range, and also 15% behind the Genie software. This drawback may be not of great importance in clinical practice, as the range limitation was only evident in the higher range and myocardium fibrosis hardly reaches these values, whereas the lower range was acceptable. Further analysis revealed the superiority of Genie software: the higher correlation with RVS, the better values in single linear regression against the reference and, most importantly, more uniform results in Bland Altman analysis. While the Colocalization software was overestimating at the lower end and underestimating at the higher end, Genie software was only slightly underestimating in a mid-range with the results still exceeding those of the pathologist’s mean score. Of note, both digital algorithms produced slightly different results, a fact that might appear surprising. Despite both algorithms are aimed to measure the same feature, namely, the proportion of connective tissue in the myocardium, they are still based on different principles and may result in different measurement errors. While Colocalization classifies each pixel according to its color characteristics, the Genie software is based on a far more complex pattern recognition system, which also refers to spatial aspects of the image. Probably, the only relevant drawback of Genie was the underestimation bias of 1.61% from the RVS. Overall, the Genie classifier performed best in our study, being closest to the RVS, with almost perfect correlation, adequate range and uniform results throughout the whole scale.

Potential limitations of the Genie software are related to the necessity to train the system to identify the various structures of interest, which is time-consuming and based on the inherent subjectivity of the “human trainer”. This fact also makes the Genie software sensitive to inter-laboratory reproducibility issues. However, after the adaptation of Genie software to the clinical needs it can be run fully automated and as a result it can be equally as time-efficient as the Colocalization software is. The Genie software has the possibility of tuning the algorithm, which makes it more flexible in practical maintenance. Even if 2% is an acceptable error for cardiac fibrosis estimate in clinical sense, this algorithm may require further adaptation to potential sources of slide quality variation.

The Colocalization software has also proved to be a fully acceptable method for cardiac fibrosis measurement. In clinical practice, the Colocalization software should provide similar precision and accuracy as the Genie tool, because cardiac fibrosis values are rarely exceeding 40%, and a slight overestimation in the lower range may be acceptable. The Colocalization software is less complex, simpler to use and calibrate, and less expensive. Furthermore it can be run fully automated from image scanning to the final results and it is very time efficient. The Colocalization algorithm is less dependent on human investigator input at any point of the process (except initial settings for color deconvolution), making it more transparent and manageable for users.

## Conclusions

Both digital image analysis methods based on colocalization and pattern recognition algorithms revealed almost perfect correlation with the criterion standard obtained by stereology grid count and, in terms of accuracy, outperformed the pathologist’s visual score. The Genie algorithm proved to be the method of choice with the single drawback of a slight underestimation bias that can be acceptable for clinical and research demands to quantify the extent of fibrosis in myocardial biopsies.

## Competing interests

The authors declare that they have no competing interests.

## Authors’ contributions

DD, JB, EZ, BP, PH, AiL and ArL drafted the manuscript. DD, JB, AiL and ArL designed and performed the digital analyses. BP and PH designed and produced the stereology grid counts. EZ performed the pathologist’s scoring of the microscopy slides. DD and ArL performed statistical analysis. All authors participated in conception and design of the study, critically reviewed the analysis results, read and approved the final manuscript.
